# The current situation of health equity in underserved areas of Afghanistan

**DOI:** 10.3389/fpubh.2024.1370500

**Published:** 2024-09-24

**Authors:** Marwa Rashad Salem, Nelly Hegazy, Sherif Eldeeb, Jerome A. Shaguy, Ramesh Mohammad Nassery, Abdullah Khawari, Jamshed Tanoli, Alaa Abouzeid

**Affiliations:** ^1^Public Health and Community Medicine, Faculty of Medicine, Cairo University, Cairo, Egypt; ^2^Public Health and Community Medicine, Faculty of Medicine, Helwan University, Cairo, Egypt; ^3^Community Medicine Research Department, National Research Centre, Giza, Egypt; ^4^Emergency Department of the WHO Organization, The World Health Organization, Kabul, Afghanistan

**Keywords:** Afghanistan, underserved areas, provinces, equity, vulnerable groups, health services

## Abstract

**Background:**

In Afghanistan, providing universal health coverage (UHC) in accordance with the principles of the UHC declaration is challenging on two fronts: the geographic topography of the country and the prevailing gender dynamics within the local culture.

**Methods:**

We conducted a desk review of underserved areas in the context of health services by gathering and analyzing existing literature, reports, and data using a combination of keywords and phrases such as: “underserved areas,” “healthcare disparities,” “access to healthcare,” and “health services.” The primary data were derived from an analysis of underserved populations conducted by the World Health Organization (WHO) Afghanistan's Emergency Program, supplemented by information from in-country partners. In addition to other reports, this review focused on analyzing the geographical availability of primary healthcare (PHC) services by employing the guidelines set forth in the SPHERE framework. It also took into account the social dynamics within the Afghan population that may create barriers to equity in terms of demand and access to PHC services.

**Results:**

Although there are a significant number of primary healthcare facilities in operation (4,242), they are unevenly distributed across different regions of Afghanistan, resulting in almost 25% of the population being underserved. The underserved population is nearly equally distributed between genders, with the majority residing in rural communities. Women of childbearing age represent 28% of the underserved population. Children under the age of five represent 16–18% of the underserved population in all regions, except in the western region, where they represent between 12 and 13%. Individuals over 60 years of age represent 1–3% of the underserved population across all regions. More than 50% of the population in the Central Highlands of Afghanistan is underserved, followed by the western and southern regions. Ghor province in the western region has the highest proportion of underserved populations, followed by Zabul province in the southern region.

**Conclusion:**

Afghanistan is currently experiencing a protracted humanitarian crisis, with millions of people living in poverty and lacking access to healthcare. This situation exposes them to serious risks such as disease epidemics, starvation, and maternal and child mortality. It is crucial to implement alternative strategies to reach the most affected populations and to increase funding for the delivery of healthcare services in Afghanistan.

## 1 Introduction

In Afghanistan, health services are provided mainly through primary healthcare (PHC) facilities (4,242), with different categories distributed in 401 districts of 34 provinces, through two key programs: the Basic Package of Health Services (BPHS) and the Essential Package of Hospital Services (EPHS) (1). In early 2021, the Integrated Package of Essential Health Services (IPEHS) was endorsed by the previous republican government to expand the coverage and scope of health services and to help move the country toward universal health coverage (UHC) (2). However, the latter was not implemented due to a lack of commitment, a lack of resources, and the country's health strategy (3).

The World Health Organization (WHO) recently completed a geospatial analysis of underserved areas in Afghanistan in September 2022; the country's health system has made significant strides in recent years, which has also improved the population's health (4). Despite these improvements, Afghanistan's health indicators are near the bottom of international indices (5) and are lower than any other country in the region. With one of the highest rates of acute malnutrition and stunting in children under the age of five worldwide, health is still of utmost importance in this nation (6). According to the United Nations Children's Fund (UNICEF), 1 million children are expected to suffer from acute severe malnutrition, while 7.4 million children in Afghanistan need humanitarian aid to survive. The mortality rate for children under the age of five is 55.7 deaths per 1,000 live births (7). Afghanistan's mountainous landscape, vulnerability to natural disasters, history of numerous conflicts over the years, and its designation as the most impoverished nation in the Asia–Pacific region in terms of the average per capita income have collectively made achieving universal access to primary healthcare extremely challenging (8, 9).

Despite the distribution of 4,242 primary healthcare facilities throughout the country, 9.5 million people still lack access to primary healthcare (underserved) (10). This finding is alarming as equitable access to primary healthcare (PHC) is a key policy concern and also a fundamental human right. The accessibility and equitable distribution of PHC are fundamental to achieving the Sustainable Development Goals (SDGs) and universal health coverage (10). The highest possible level of health and wellbeing for people can be achieved through PHC. Defined by the World Health Organization and UNICEF, PHC is a comprehensive approach that focuses on people's needs, promoting health, preventing diseases, and providing access to treatment, rehabilitation, and palliative care (11).

According to the World Health Organization, universal health coverage “means that all people have access to the full range of quality health services they need, when and where they need them, without financial hardship.” It covers the full continuum of essential health services, from health promotion to prevention, treatment, rehabilitation, and palliative care across the life course (12).

In Afghanistan, providing universal health coverage in accordance with the principles of UHC declaration is challenging on two fronts: the geographic topography of the country and the prevailing gender dynamics within the local culture. Nonetheless, a quantum of resources and a high level of effort have been expended in implementing universal health coverage. This study aimed to review the level of primary healthcare penetration into the most vulnerable and hard-to-reach populations in Afghanistan. This review article focused on analyzing the geographical availability of PHC services by employing the guidelines set forth in the SPHERE framework. It also took into account the social dynamics within the Afghan population that may create barriers to equity in terms of demand and access to PHC services.

## 2 Methods

We conducted a desk review on underserved areas in the context of health services by gathering and analyzing existing literature, reports, and data to provide a comprehensive overview of the issue. The collected data were analyzed using a descriptive approach. This approach aimed to provide a detailed and itemized description of the social conditions of vulnerable populations in Afghanistan and to identify how these conditions intersect with the ability to either demand or access the health services provided. The gathered data—all of it secondary—were screened by the research team using a systematic approach. This approach involved data display and line-by-line and keyword associations to identify themes. This approach was considered the most rigorous, providing the level of granularity and contextual grounding necessary for examining socioeconomic issues.

**Figure 1:**
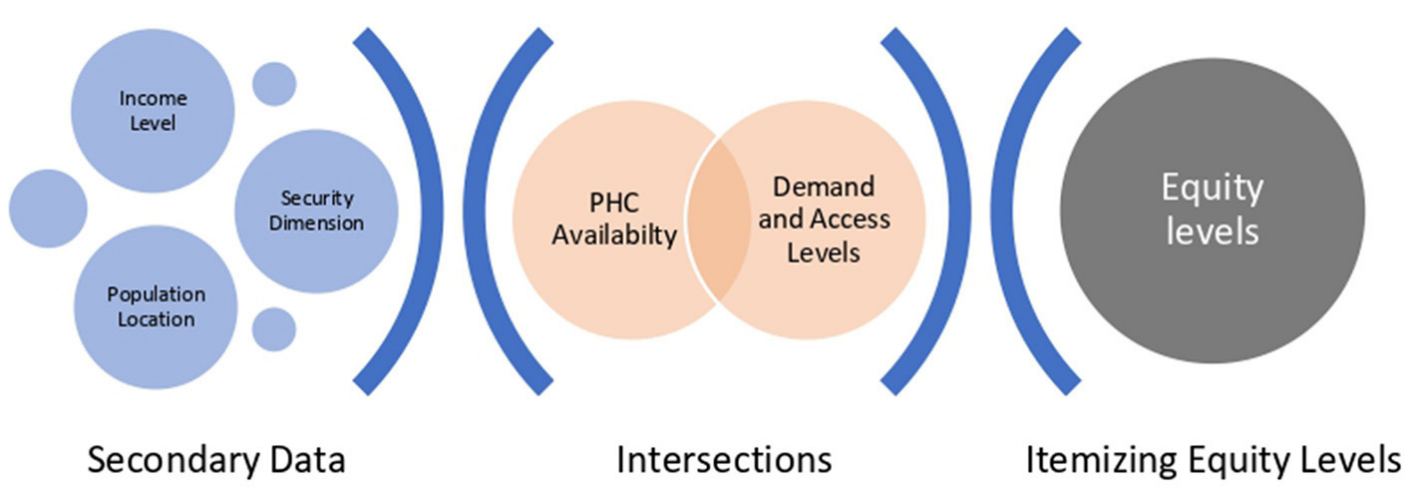
Illustrating the study methodology.

There were two stages in the review process:

1) **Initial literature search**: The systematic search process helped formulate the inclusion criteria, which included the following: the date of publication, the location of the study, and peer-reviewed papers and data. The following search keywords and phrases related to the current research question were used: “underserved areas,” “healthcare disparities,” “access to healthcare,” “health services,” and other relevant terms. The primary data were derived from an analysis of underserved populations conducted by the WHO Afghanistan's Emergency Program, supplemented by information from in-country partners. In addition to a total of 20 publications, the following were included: the underserved population analysis, which is a geographic illustration published in 2022, the Humanitarian Development Report (23), the WHO Emergency Operational Plan (24), addressing the health needs of Afghanistan crisis-affected populations, and key Afghanistan demographic indicators (based on UNICEF's 2023 data). These data were collated, carefully reviewed, and subsequently analyzed for quality in the next stage.
2) **Paper review and analysis:** The research team leader divided the documents among seven team members. Each member reviewed the documents that best aligned with their core training and backgrounds. Then, the team members organized and presented the data using simple Excel spreadsheets. This display process enabled investigators to identify themes and patterns, which corresponded to the study's goal of understanding the factors that affect vulnerable populations and the level of choice and agency these populations have in accessing primary healthcare. The analytical approach in this study was thematic, involving scheming and reading through data sources to reveal common areas that showed a convergence of issues. This process, in turn, validated them and, by extension, demonstrated rigor and robustness.
By reviewing, cross-validating, and analyzing the data and reports, this review determined the underlying dynamics in terms of social determinants and how they affect universal health coverage. It focuses on population access and the ways to improve healthcare and outcomes. The collected data and information were analyzed in iterative and interconnected steps (as shown in Figure 1).

## 3 Results

**Figure 2:**
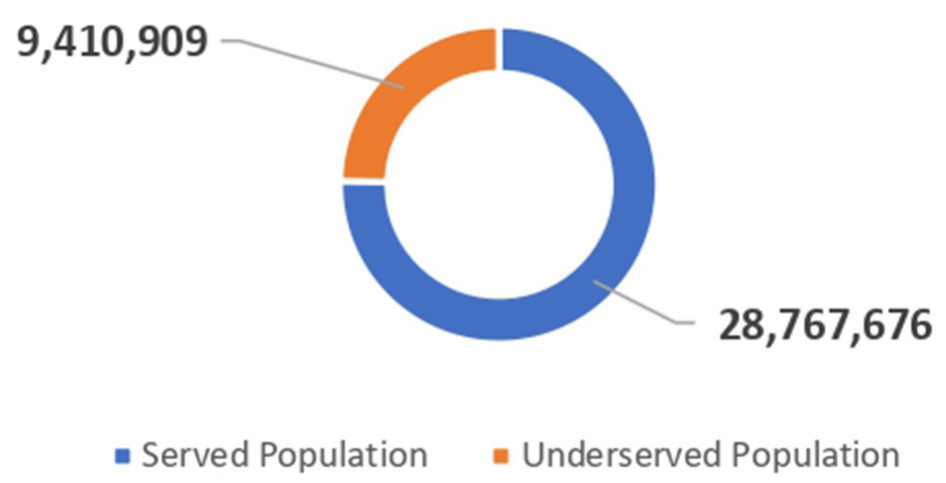
The pie chart represents the total served population vs. the total underserved population in Afghanistan.

The total population of Afghanistan is ~38.2 million (10). However, 9.4 million people are deprived of primary healthcare services (Figure 2). The underserved population is nearly equally distributed between genders, with the majority of them residing in rural communities (Table 1).

**Table 1:** Distribution of the studied underserved population by gender and residence (*n* = 9,410,909).

**Characteristics**	**No**.	**%**
**Gender**		
Men	4,766,863	50.6
Women	4,644,046	49.4
**Residence**		
Urban	1,007,662	10.7
Rural	8,403,247	89.3

As shown in Table 2, the available health services are predominantly provided through primary healthcare facilities (4,242), among which the sub-health center and the basic health center are the most prevalent. The Health Emergency Response (HER) Project, which is funded by the World Bank, supports more than half (55%) of the health facilities in Afghanistan.

**Table 2:** Percentage distribution of health facilities by type and category in Afghanistan (*n* = 4,242).

**Types of health facilities**	**No**	**%**
Sub health center	1,286	30.3
Basic health center	1,003	23.6
Mobile health team	720	17.0
Comprehensive health center	451	10.6
Family health house	320	7.5
District hospital	94	2.2
First aid trauma post	92	2.1
Provincial hospital	32	0.7
Specialized hospital	27	0.6
Regional hospital	16	0.4
Notational hospital	5	0.1
Health posts and community health providers	196	4.6
**Categories of health facility**		
HER facilities	2,296	54.1
Non-HER health facilities	1,946	45.9

HER, Health Emergency Response.

Tables 3–5 show the percentage distribution of the underserved populations in 34 provinces of Afghanistan. Although there are a significant number of health services in operation (4,242), they are unevenly distributed across different regions of Afghanistan, resulting in 25% of the population being underserved. Women of childbearing age (15–49 years) represent 28% of the underserved population in the whole of Afghanistan. Children under the age of five represent 16–18% of the underserved population in all regions, except in the western region, where they represent between 12 and 13%. Individuals over 60 years of age represent 1–3% of the underserved population across all regions.

**Table 3:** Percentage distribution of the underserved population in the capital and Central Highlands of Afghanistan by gender and vulnerable age groups (*n* = 10,362,087).

**Region**	**Total population**	**Underserved population**										
		**% of underserved population** ^*^	**Men**		**Women**		**Women in the childbearing age group (15–49 years)**		**Children under the age of five**		**Over the age of 60**	
	**No**	**%**	**No**	**%**	**No**	**%**	**No**	**%**	**No**	**%**	**No**	**%**
**Capital**												
Logar	568,537	11.98	33,697	49.0	34,421	51.0	32,280	47.0	11,148	16.0	1,982	3.0
Parwan	747,063	11.77	49,254	49.0	50,426	51.0	47,243	47.0	16,262	16.0	2,901	3.0
Panjsher	196,255	23.37	22,712	50.0	23,163	50.0	21,738	47.0	7,525	16.0	1,335	3.0
Kabul	6,060,065	7.6	226,067	49.0	236,804	51.0	219,633	47.0	73,095	16.0	13,470	3.0
Kapisa	598,752	12.24	36,273	49.0	37,049	51.0	34,746	47.0	12,001	16.0	2,134	3.0
Maidan Wardak	916,376	36.96	167,810	50.0	170,939	50.0	160,507	47.0	55,656	16.0	9,858	3.0
**Central Highlands**												
Daykundi	629,387	50.73	156,181	49.0	163,163	51.0	153,718	48.0	51,056	16.0	7,121	2.0
Bamyan	645,652	54.62	172,330	49.0	180,380	51.0	169,567	48.0	55,912	16.0	7,865	2.0

^*^Out of the total population.

**Table 4:** Percentage distribution of the underserved population in eastern, northeastern, southeastern, and western regions of Afghanistan by gender and vulnerable age groups (*n* = 18,230,474).

**Region**	**Total population**	**Underserved population**										
		% of underserved population*	**Men**		**Women**		**Women in the childbearing age group (15–49 years)**		**Children under the age of five**		**Over the age of 60**	
	**No**	**%**	**No**	**%**	**No**	**%**	**No**	**%**	**No**	**%**	**No**	**%**
**Eastern**												
Laghman	687,838	8.6	29,986	50.0	29,782	50.0	27,332	46.0	10,031	17.0	580	1.0
Nangarhar	2,231,919	0.58	6,563	50.0	6,499	50.0	6,036	46.0	2,212	17.0	127	1.0
Nuristan	253,621	53.43	6,563	50.0	6,499	50.0	6,036	46.0	2,212	17.0	127	1.0
Kunar	604,793	25.39	76,936	50.0	76,638	50.0	69,523	45.0	25,556	17.0	1,490	1.0
**Northeastern**												
Baghlan	1,172,694	35.7	210,641	50.0	208,013	50.0	184,627	44.0	66,510	16.0	7,661	2.0
Takhar	1,439,898	29.9	216,819	50.0	214,110	50.0	190,105	44.0	68,403	16.0	7,886	2.0
Kunduz	1,093,187	13.09	72,007	50.0	71,104	50.0	63,205	44.0	22,655	16.0	2,619	2.0
Badakhshan	1,506,936	53.8	408,233	50.0	403,161	50.0	357,481	44.0	129,203	16.0	14,849	2.0
**Southeastern**												
Paktika	599,842	34.68	108,504	52.0	99,558	48.0	88,907	43.0	37,333	18.0	4,557	2.0
Khost	1,033,390	19.0	101,659	52.0	94,763	48.0	86,511	44.0	34,132	17.0	4,302	2.0
Paktya	832,085	30.32	130,814	52.0	121,532	48.0	110,439	44.0	44,153	17.0	5,526	2.0
Ghazni	1,084,813	30.8	173,474	52.0	161,064	48.0	146,233	44.0	58,611	18.0	7,326	2.0
**Western**												
Hirat	3,058,033	29.57	466,036	52.0	438,454	48	461,936	51.0	112,065	12.0	17,999	2.0
Ghor	1,065,138	67.11	370,072	52.0	344,767	48	352,836	49.0	93,048	13.0	14,225	2.0
Badghis	784,328	51.93	210,856	52.0	196,471	48	201,174	49.0	52,976	13.0	8,106	2.0
Farah	781,959	52.05	210,221	52.0	196,831	48.0	204,472	50.0	51,683	13.0	8,100	2.0

*Out of the total population.

**Table 5:** Percentage distribution of the underserved population in northern and southern regions in Afghanistan by gender and vulnerable age groups (*n* = 9,486,347).

**Region**	**Total population**	**Underserved population**.										
		**Total underserved** ^*^	**Men**		**Women**		**Women in the childbearing age group (15–49 years)**		**Children under the age of five**		**Over the age of 60**	
	**No**	**%**	**No**	**%**	**No**	**%**	**No**	**%**	**No**	**%**	**No**	**%**
**Northern**												
Balkh	1,617,682	13.9	111,297	49.0	114,042	51.0	103,028	46.0	34,295	15.0	5,949	3.0
Samangan	548,643	54.03	146,327	49.0	150,110	51.0	129,662	44.0	48,471	16.0	7,826	3.0
Faryab	1,483,816	52.1	245,317	49.0	251,700	51.0	216,009	43.0	82,061	17.0	13,121	3.0
Jawzjan	622,253	28.49	87,548	49.0	89,767	51.0	79,017	45.0	28,160	16.0	4,681	3.0
Sar-E-Pul	737,712	34.05	124,002	49.0	127,241	51.0	108,757	43.0	41,731	17.0	6,633	3.0
**Southern**												
Zabul	413,227	60.7	127,019	51.0	123,903	49.0	111,091	44.0	44,493	18.0	1,506	1.0
Hilmand	1,312,844	39.64	263,325	51.0	257,207	49.0	230,377	44.0	92,620	18.0	3,123	1.0
Uruzgan	685,665	47.2	163,843	51.0	160,067	49.0	143,349	44.0	57,664	18.0	1,943	1.0
Kandahar	1,851,157	19.3	181,997	51.0	175,462	49.0	158,734	44.0	61,440	17.0	2,145	1.0
Nimroz	213,348	39.7	43,330	51.0	41,394	49.0	37,710	45.0	14,204	17.0	508	1.0

^*^Out of the total population.

**Figure 3:**
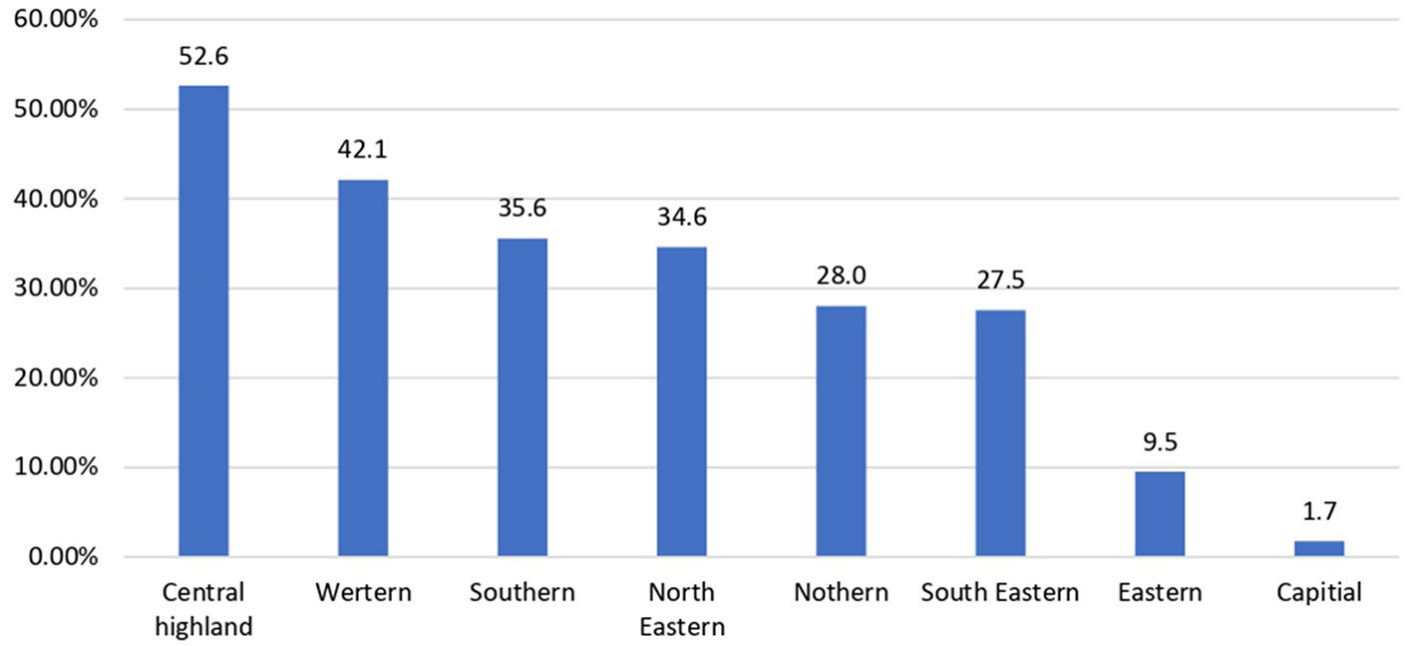
The bar chart represents the percentage distribution of the underserved areas by region in Afghanistan.

Figure 3 shows that more than 50% of the population of the Central Highlands of Afghanistan is underserved, followed by the western region at 42.1% and the southern region at 35.5%. This finding is in contrast with the capital region, which demonstrates the lowest percentage in White areas (1.7%), and the Eastern region (9.5%).

**Figure 4:**
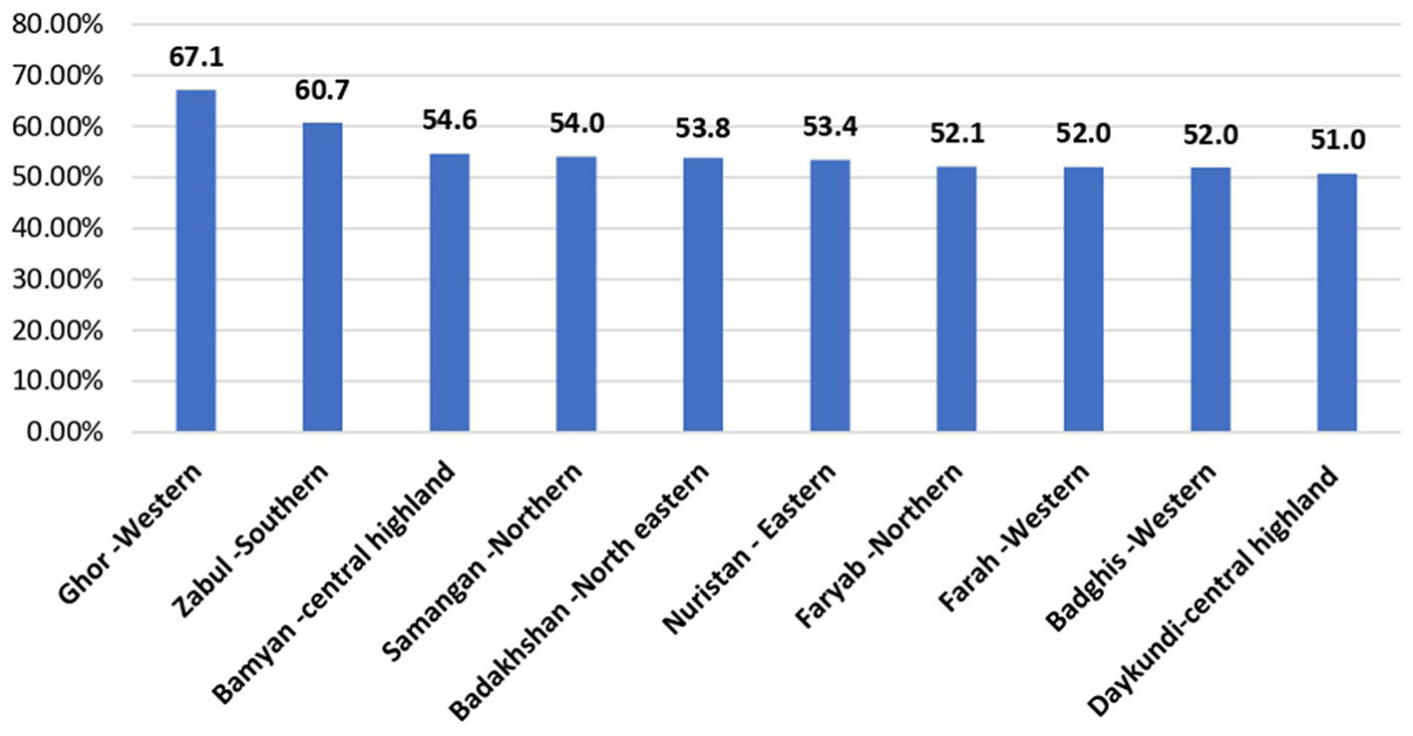
The bar chart represents the top 10 provinces demonstrating the high percentage of underserved populations in Afghanistan.

Ghor province in the western region has the highest percentage of underserved populations (67.1%), followed by Zabul province in the southern region (60.7%) and Bamyan province in the Central Highlands (54.6%). Almost the entire population in Nangarhar province in the eastern region is provided with primary healthcare services, while only 7.6% of the population of Kabul province is underserved (Figure 4).

## 4 Discussion

The present study aimed to highlight the current situation of underserved areas in Afghanistan (as a percentage of the total population, highly ranked regions, and provinces) and demonstrate the characteristics of the highly affected groups in the underserved areas by disaggregating the data of the underserved areas, based on genders and vulnerable age groups to identify the most vulnerable groups in these areas.

Approximately one-quarter of the population living in Afghanistan is deprived of any primary healthcare services. In our study, among those deprived, almost no differences were observed based on gender, and the majority (~90%) were found to reside in rural areas. The latter finding may raise major concerns and may have consequences for the health of the population, especially in a developing country with instability. The high percentage of the underserved population could be attributed to the fewer number of healthcare workers (HCWs) in rural areas compared to urban areas (13), where it is estimated that, for every 10,000 individuals, there are 17 HCWs in rural areas compared to 36 in urban areas (1). Furthermore, the gender of HCWs aggravates this problem as it is not culturally acceptable for a female patient to be examined by a male HCW (14). This is crucial considering that the United Nations Development Program has reported the rate of development to have taken slow progress, which exposes the country to insecurities and unstable conditions that affect the health status of the citizens (15).

Approximately half of the proportion of the services is delivered through either basic or sub-health centers. Providing basic health services is crucial for maintaining the health status of individuals, yet it should be also noted that only 2.2% of the services are delivered through a specialist or at least a secondary level of care (district hospital) with < 1% providing specialized care. Moreover, emergency or first aid trauma services are delivered by 2% of the health facilities. The latter two figures are of extreme value in areas of conflict. Afghanistan is characterized by having more than 80% of the land as mountainous. This aspect forces the population to be distributed or scattered over multiple small terrains (9), with a high proportion of them being mobile population (16). This is why the presence of mobile health teams or clinics delivering services is also of high value, which accounts for 17% in the present study. It should also be noted that over half of the services delivered through HER facilities rely mainly on funds from the World Bank, which can expose the country to major health consequences in case of new epidemics or catastrophes necessitating shifting of the fund.

The most affected region in the country is the Central Highlands, characterized by rugged and mountainous terrain that makes travel challenging and hinders the construction of reliable transportation infrastructure, thereby limiting access to health facilities. Even though the two provinces of this region—Daykundi and Bamyan—are not highly affected, the absence of variation could indicate that the entire region is affected in the same way. Furthermore, there are minor differences between the population of men, women, and women of childbearing age (~50%). This is different from the provinces of the western region, which fall in the second place among the overall underserved regions. It is the highest in Ghor (67%), whereas it is the lowest in Hirat, which was below 30%. The latter figure indicates that the variations within provinces require different approaches to adjust and apply. In the southern region—the third most affected one—the variation is much greater, being highest in Zabul and lowest in Kandahar.

The capital region has the lowest percentage of the underserved population, with Kabul having the lowest and Maidan Wardak province the highest, showing nearly a 30% variation in the percentage. Nangarhar province, in the eastern region, has the lowest underserved population (< 1%). However, as in most regions, there are wide variations between provinces in the eastern region. These disparities between different regions and provinces could be attributed to many factors, such as poverty, transportation, roads, and areas of previous conflicts (16). A high percentage of out-of-pocket health expenditures alongside poverty presents a major challenge to the access to health services, which results in the population being underserved (17). Moreover, in early 2000, approximately half of the respondents in a survey reported not seeking treatment for health problems (18). The presence of transportation and well-constructed roads were the key factors leading to contrasting figures. In Kabul, the capital, more than 50% of the population had access to transport for emergency obstetric facilities; however, this figure decreased to < 20% in Badakhshan province in the far northeast (13). Furthermore, harsh weather conditions, including snowfall and flooding, can augment the problem of internally displaced people, disrupt transportation, and make it difficult for individuals to reach health facilities, especially during the winter months in some parts of the country (19). In addition, unstable conditions, such as war or conflicts, are also responsible for the inability to provide citizens with health services. In areas of conflict, such as Helmand, the health facilities have dropped by more than 35% owing to unstable conditions during the years 2004–2006 (20). Furthermore, challenges related to the presence of landmines and unexploded ordnance, particularly in previous conflict-affected areas, can pose a threat to individuals seeking healthcare and limit the development of infrastructure (21).

Minimal overall differences between men and women were also present within each region and province. This finding could indicate that the lack of access to primary or any health service is not primarily based on gender discrimination. Moreover, women of childbearing age show similar patterns, yet this group is highly vulnerable. The latter group represents a major challenge in recruiting female HCWs because the problem of unmet needs exists despite multiple efforts (22).

The region with the highest underserved older adult population is the northern region, with the same percentage (3%) in all provinces of this region. However, it does not have the highest percentage of underserved populations of children under the age of five. On the other hand, southern and southeastern regions have the highest percentages of underserved populations of children under the age of five. This could require different approaches to provide these age groups with suitable services. The group comprising children under the age of five is a vulnerable one, especially with an estimated mortality rate of 55.7 deaths per 1,000 live births and with approximately one million children experiencing symptoms associated with malnutrition [(7) in the background].

## 5 Study limitations

This study employed robust methodologies, gathering the most reliable and recent data on the social and economic situation in Afghanistan. Additionally, the data on the presence of primary healthcare services were also collated. However, this study was conducted within the constraints of the available population data. Primary data on Afghanistan are generally hard to find, and most comprehensive surveys are too old and outdated to be considered credible. Furthermore, data sources relating to Afghanistan are often not open-source, i.e., they are not accessible. These limitations are acknowledged in relation to the findings of the study.

## 6 Conclusion

Afghanistan is currently experiencing a protracted humanitarian crisis, which is impacting millions of people living in poverty or lacking access to healthcare. Approximately 9.4 million Afghans lack access to primary healthcare services, with the majority residing in rural communities. The Central Highlands, western region, and southern region exhibit the highest rates of underserved populations. Notably, Ghor province in the western region and Zabul province in the southern region stand out as having the most significant number of individuals without access to primary healthcare. Furthermore, the analysis highlights the vulnerabilities of women of childbearing age, as well as children under the age of five, who comprise a considerable portion of the underserved population. This situation elevates the risk of disease outbreaks, famine, and heightened mortality, particularly among the vulnerable populations. The status is further exacerbated by ongoing conflicts, extreme weather events, and the legacy of decades of instability, which have significantly weakened the healthcare system. Addressing this crisis requires a multi-pronged approach that prioritizes increasing funding for healthcare services, implementing alternative strategies to reach the most vulnerable populations, and ensuring equitable access to healthcare across the nation, particularly in under-resourced and marginalized communities.
